# Treatment of Ununited Fractures

**Published:** 1861-05

**Authors:** J. C. Stone

**Affiliations:** Iowa City


### TREATMENT OF UNUNITED FRACTURE.

IOWA CITY, April 10th, '61.

PROF. BRAINARD—*Dear Sir* :—I have the satisfaction this morning of adding another link to the chain of testimony in favor of your mode of treating ununited fracture.

The man Dwire, with ununited fracture of humorus, I have this morning "turned out" with a sound bone.

There is, (as we always expect in such cases), much emaciation and want of muscular motion, in the affected arm, which a few weeks, with his general good health, will rectify.

Yours in haste, J. C. STONE.

---

The above case was one of fracture of the humerus, of over a year's standing, in which exsection had already failed according to the statement of the patient. It was therefore very unfavorable. [B.]

